# Supplementary material for: Ultrathin metal–organic framework membrane production by gel–vapour deposition
Source: Nat Commun. 2017 Sep 1;8:406. doi: 10.1038/s41467-017-00544-1 (PMC5581339; doi:10.1038/s41467-017-00544-1)
Supplement: Supplementary file 1 — Supplementary Information [file 41467_2017_544_MOESM1_ESM.pdf]

Description of Supplementary Files

File Name: Supplementary Information
Description: Supplementary Figures, Supplementary Tables and Supplementary References

File Name: Peer Review File

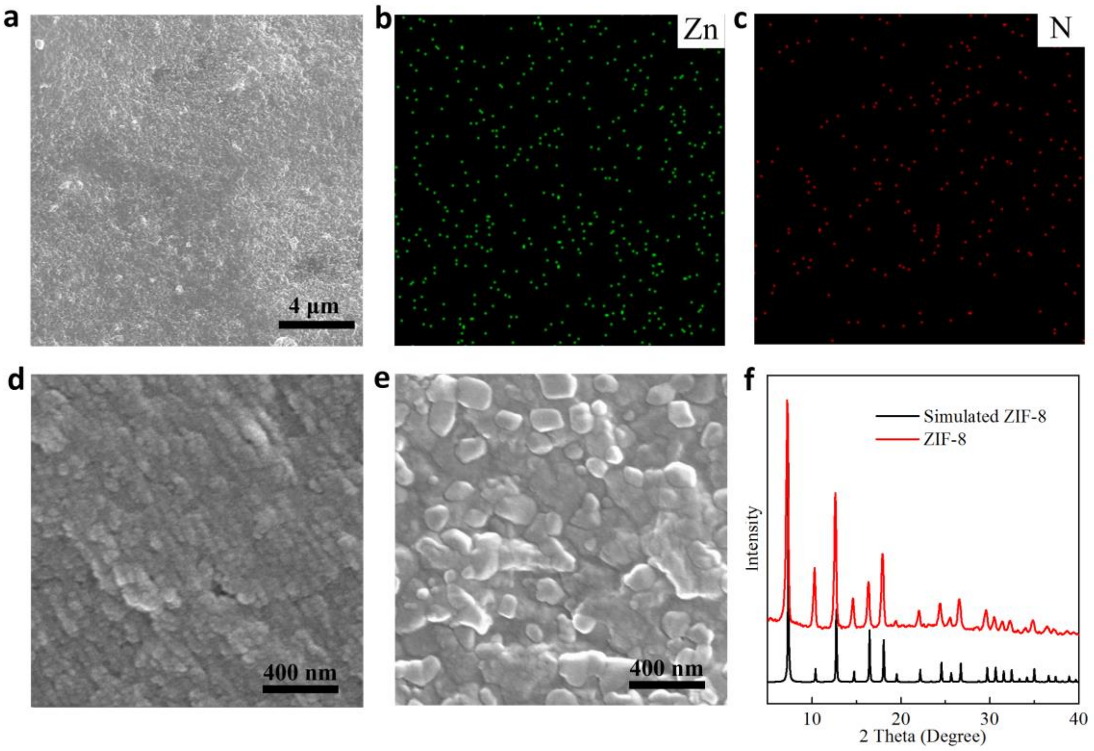

Supplementary Figure 1 | SEM images and XRD patterns of ZIF-8 membranes. **a**, Top view SEM image of ZIF-8 membrane. The sample was prepared with sol concentration of 1 U, coating time of 2 s and deposition time of 2 h. **b**,**c**, Energy dispersion spectroscopy (EDS) mapping of the ZIF-8 membrane corresponding to image **a**. Top view SEM image of **d**, the inner surface and **e**, the ZIF-8 layer on inner surface of the PVDF hollow fibre. **f**, XRD patterns of the simulated ZIF-8 and the ZIF-8 membrane on inner surface of the PVDF hollow fibre.

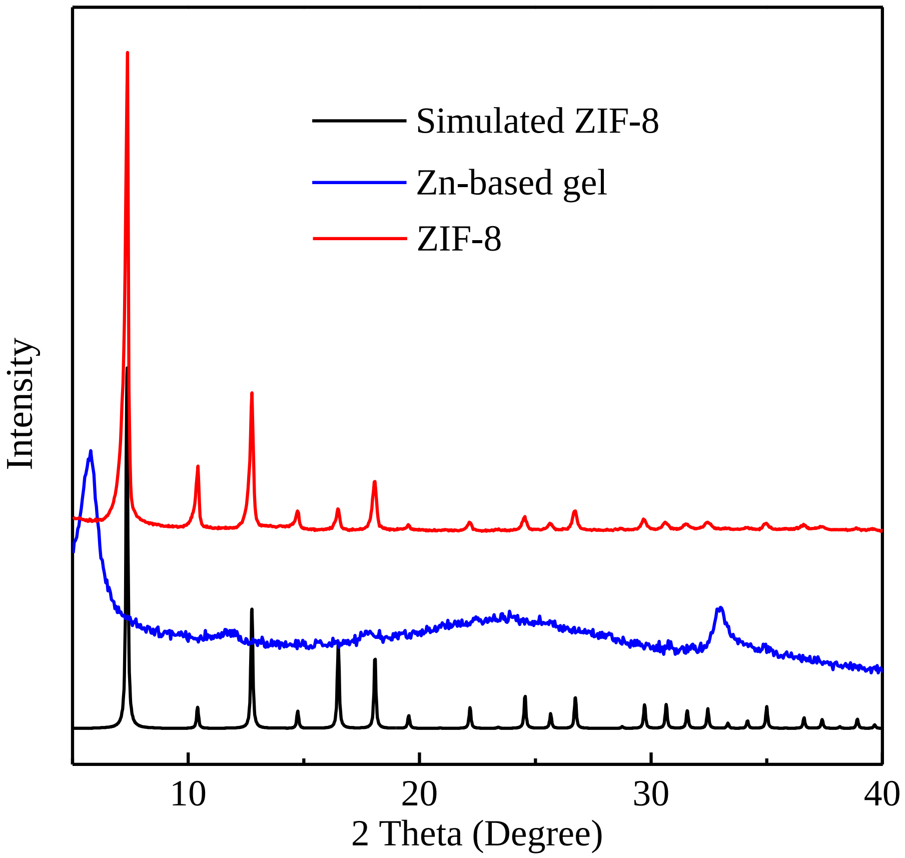

Supplementary Figure 2 | Powder XRD patterns of Zn-based gel and experimental ZIF-8 powder. The Zn-based gel and bulk ZIF-8 powder were synthesized without substrate. The simulated XRD pattern of ZIF-8 is shown for reference. This result indicates that gel has been transformed to ZIF-8, and the prepared ZIF-8 shows intact crystalline structure.

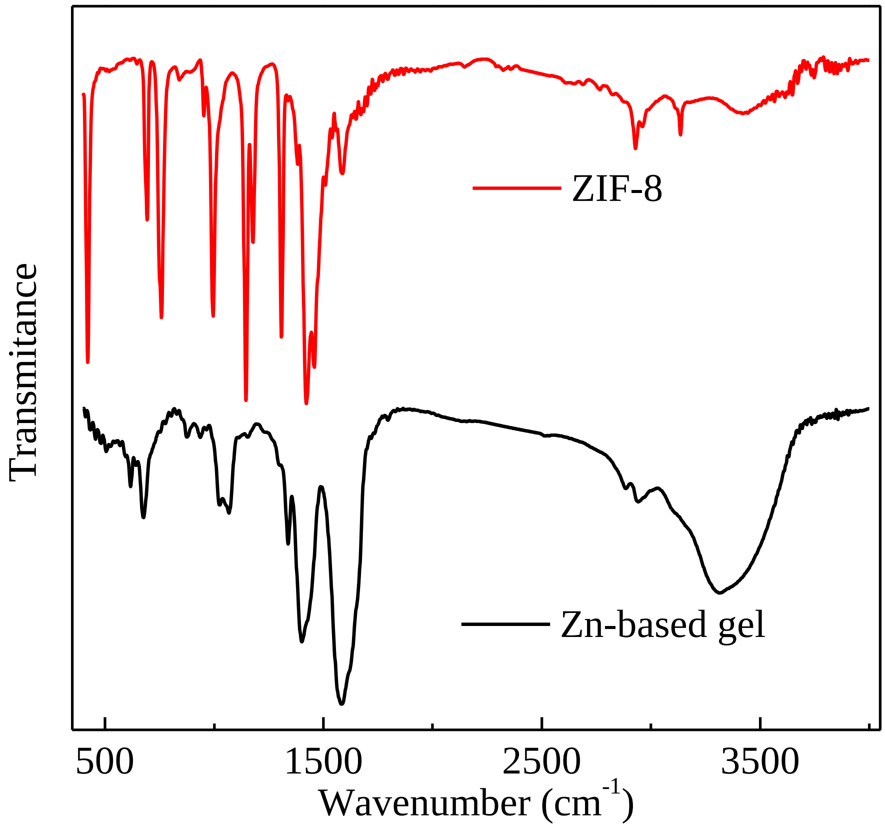

Supplementary Figure 3 | FTIR spectrums of Zn-based gel and prepared ZIF-8. The Zn-based gel and bulk ZIF-8 powder were synthesized without substrate. This result shows the change of chemical structure after vapour deposition, demonstrates the formation of ZIF-8.

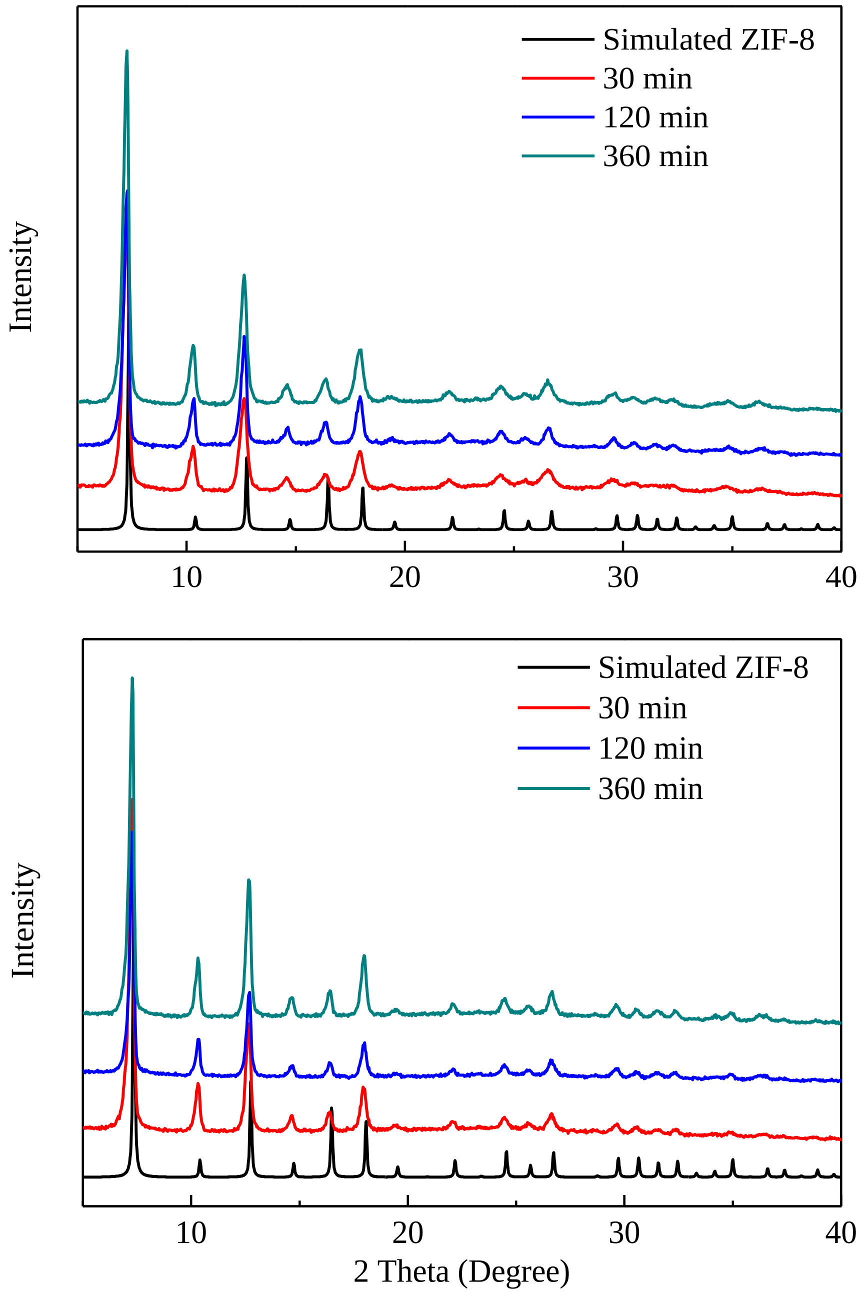

Supplementary Figure 4 | XRD patterns of ZIF-8 membranes with different deposition time (top) and calcinated ZIF-8 membranes with different deposition time (bottom). The simulated XRD pattern of ZIF-8 is shown for reference. There are no characteristic peaks of ZnO in calcinated ZIF-8 membranes, which verifies that all Zn-based gel has been transformed to ZIF-8.

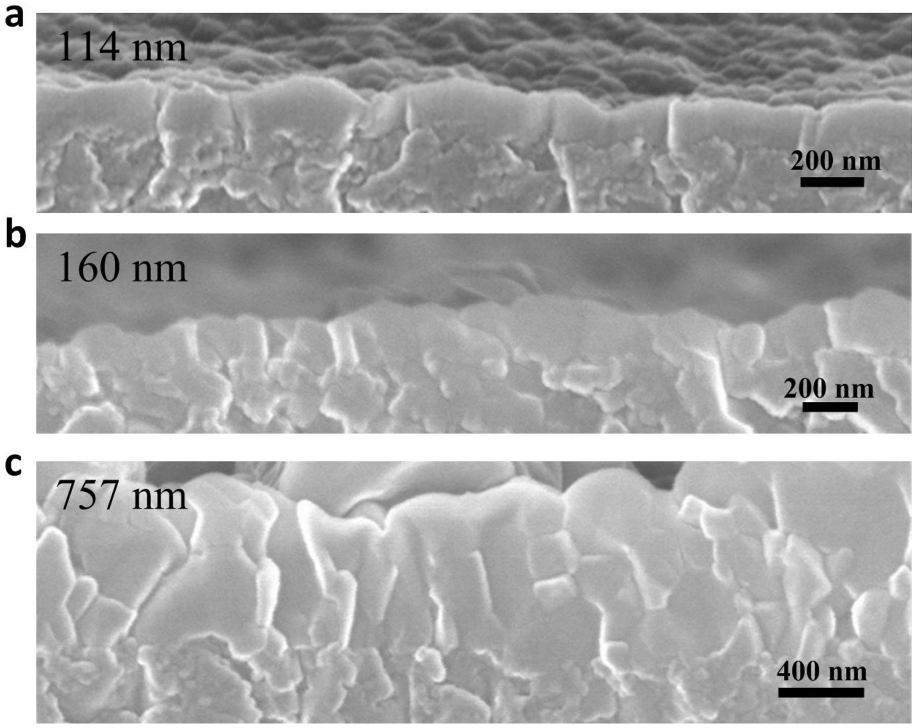

Supplementary Figure 5 | Cross-sectional view SEM images of ZIF-8 membranes with various coating times. **a**, 5 s, **b**, 10 s and **c**, 30 s. These ZIF-8 membranes were prepared with sol concentration of 1 U and deposition time of 2 h. The thickness of the membrane increases with the coating time.

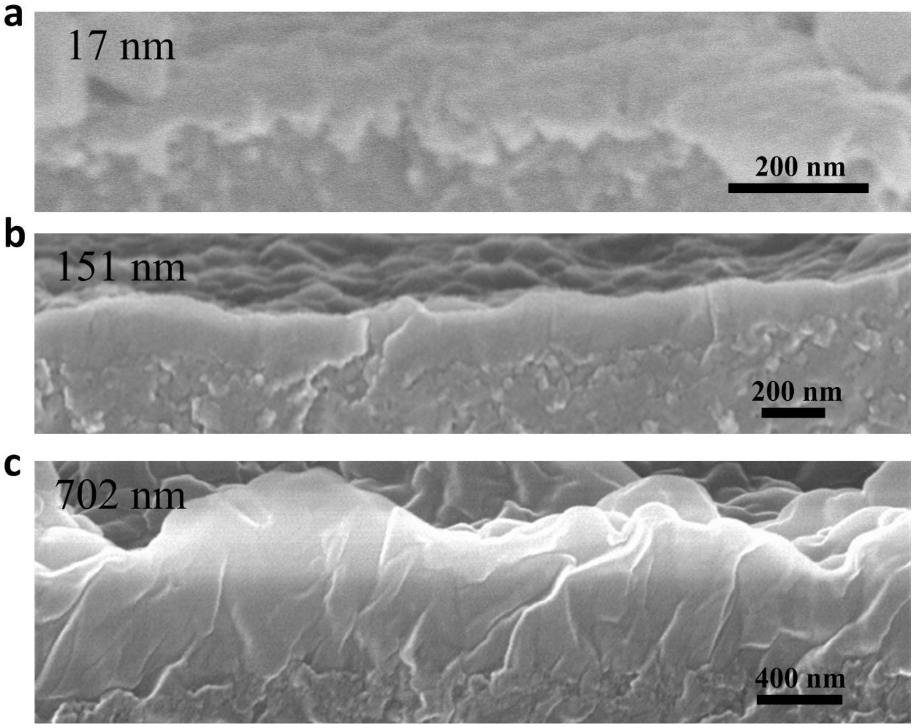

Supplementary Figure 6 | Cross-sectional view SEM images of ZIF-8 membranes with various sol concentrations. **a**, 0.1 U, **b**, 1.5 U and **c**, 2 U. These ZIF-8 membranes were prepared with coating time of 2 s and deposition time of 2 h. The thickness of membrane increases with the sol concentration.

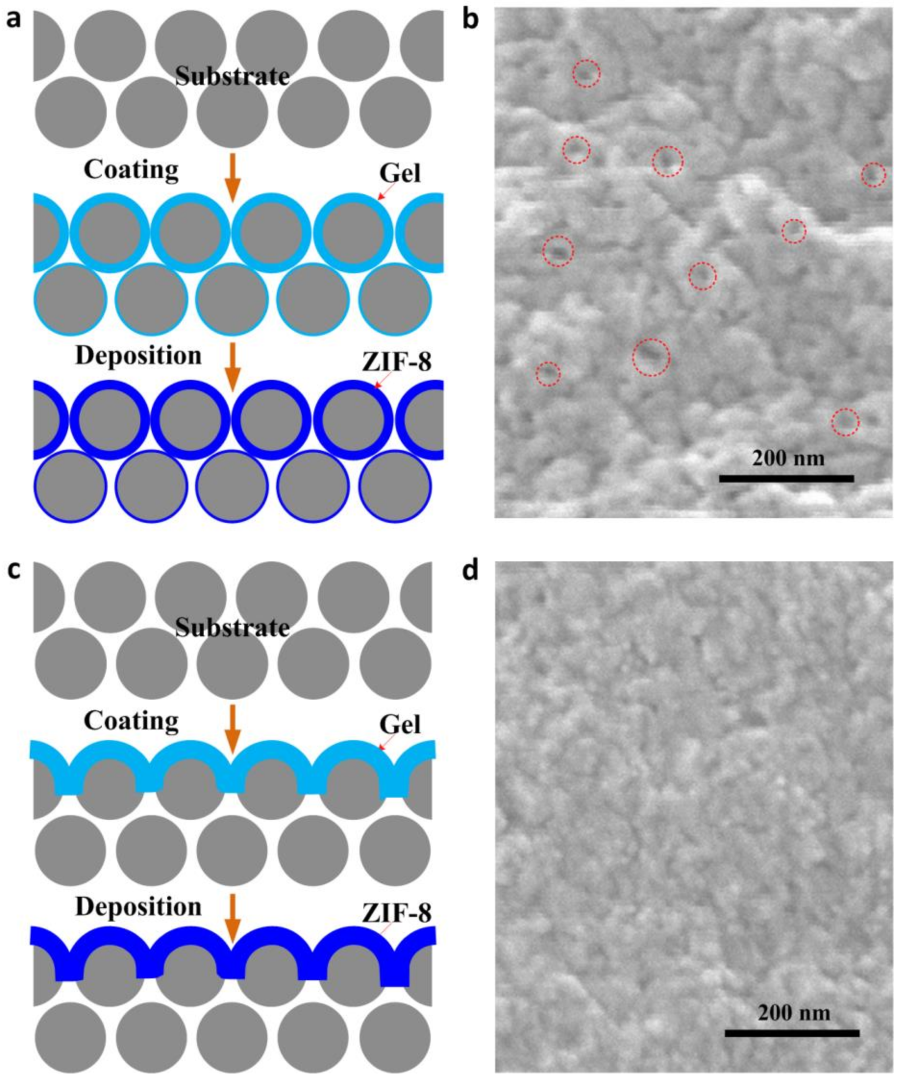

Supplementary Figure 7 | Schematic diagrams and top view SEM images. a,**c**, Schematic diagram and **b**,**d**, top view SEM image of **a**,**b**, the noncontinuous ZIF-8 membrane fabricated with sol concentration of 0.05 U and **c**,**d**, the continuous ZIF-8 membrane fabricated with sol concentration of 0.1 U. These ZIF-8 membranes were prepared with coating time of 2 s and deposition time of 2 h. The small concentration means the low viscosity, which leads to the small loading and spread in internal substrate of Zn-based sol. Because the surface of porous substrate is composed of nanoparticles with diameter of 20-50 nm, the formed membrane has some cracks.

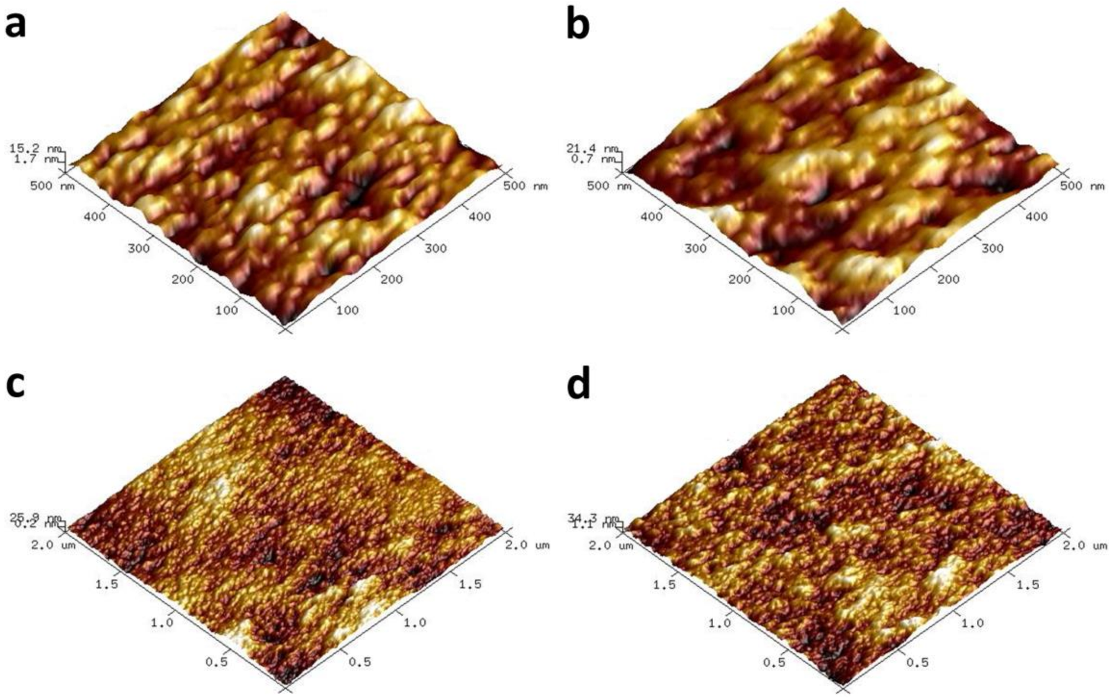

Supplementary Figure 8 | AFM images of Zn-based gel layer and ZIF-8 membrane. AFM analysis of **a**,**c**, Zn-based gel layer and **b**,**d**, ZIF-8 layer on PVDF hollow fibres. The samples were prepared with sol concentration of 0.1 U and coating time of 2 s. The vapour deposition time was 2 h. Root mean square roughness (R_q): **a**, 3.6 nm, **b**, 5.8 nm **c**, 7.0 nm, **d**, 9.3 nm. Arithmetic average roughness (R_a): **a**, 2.8 nm, **b**, 4.7 nm **c**, 5.5 nm, **d**, 7.3 nm.

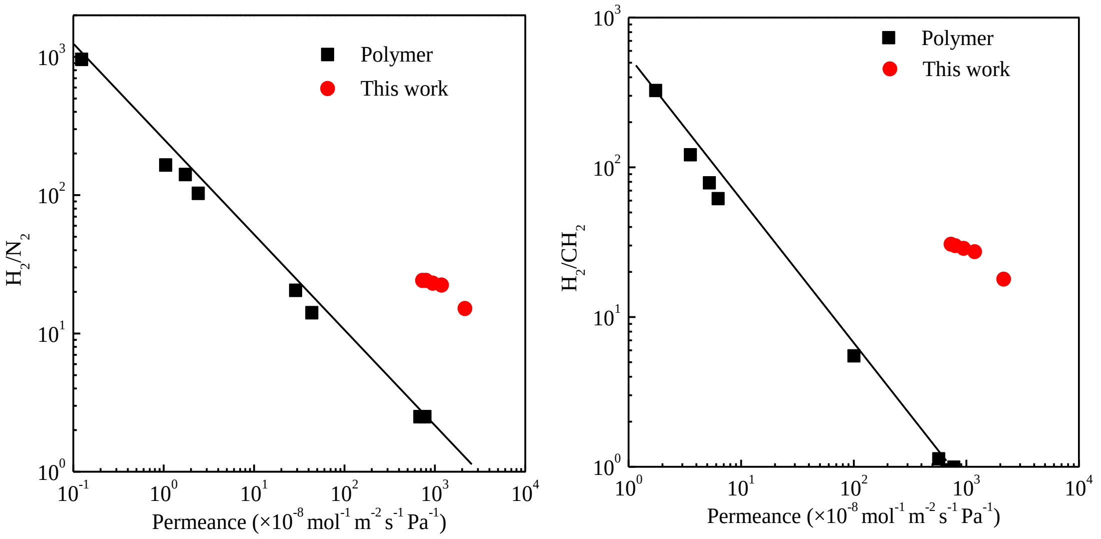

Supplementary Figure 9 | Comparison of the prepared ZIF-8 membranes with polymer membranes reported in previous studies for H_2/N_2 and H_2/CH_4 systems. The black line is the Robeson's upper-bound of polymeric membranes reported in 2008. The permeance is calculated from permeability by assuming membrane thickness of 1 μm. In H_2/N_2 and H_2/CH_4 systems, the performance of our membranes can surpass the Robeson's upper-bound.

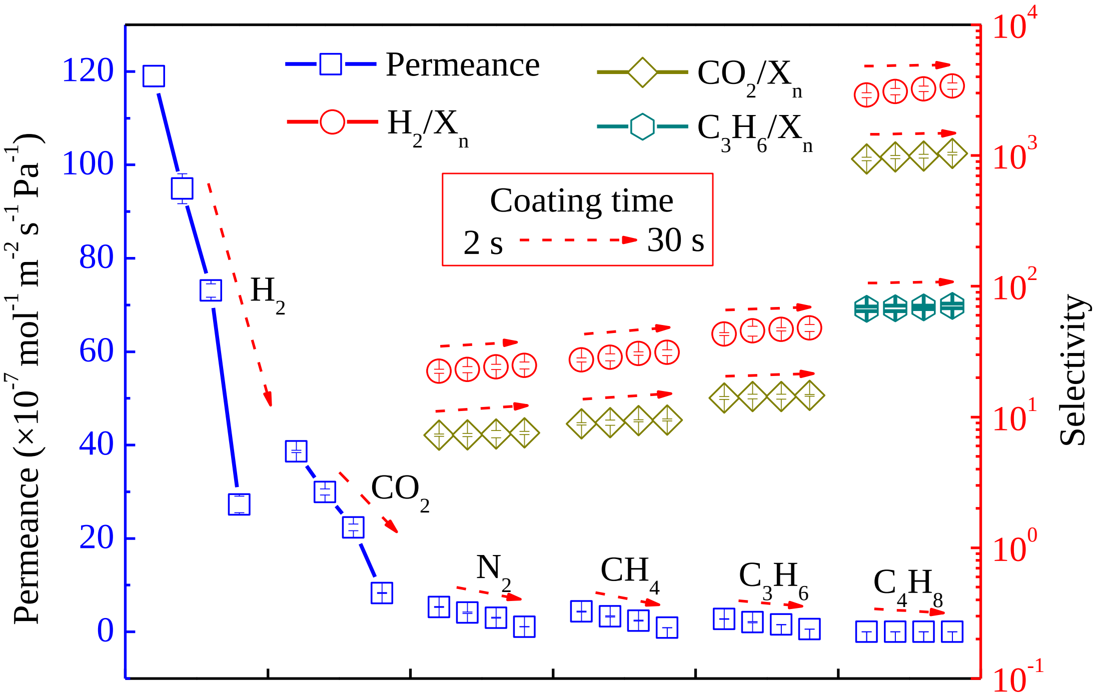

Supplementary Figure 10 | Single gas permeation behaviour of various gases through ZIF-8 membranes with various coating times of 2 s, 5 s, 10 s and 30 s. These ZIF-8 membranes were prepared with sol concentration of 1 U and deposition time of 2 h. Blue square is gas permeance. H_2/X_n, CO_2/X_n and C_3H_6/X_n selectivities are depicted in red circle, dark yellow rhombus and dark cyan hexagon, respectively. X_n represents various gases. Red arrow shows the increasing direction of coating time. Gas permeances decreased with increases of coating time.

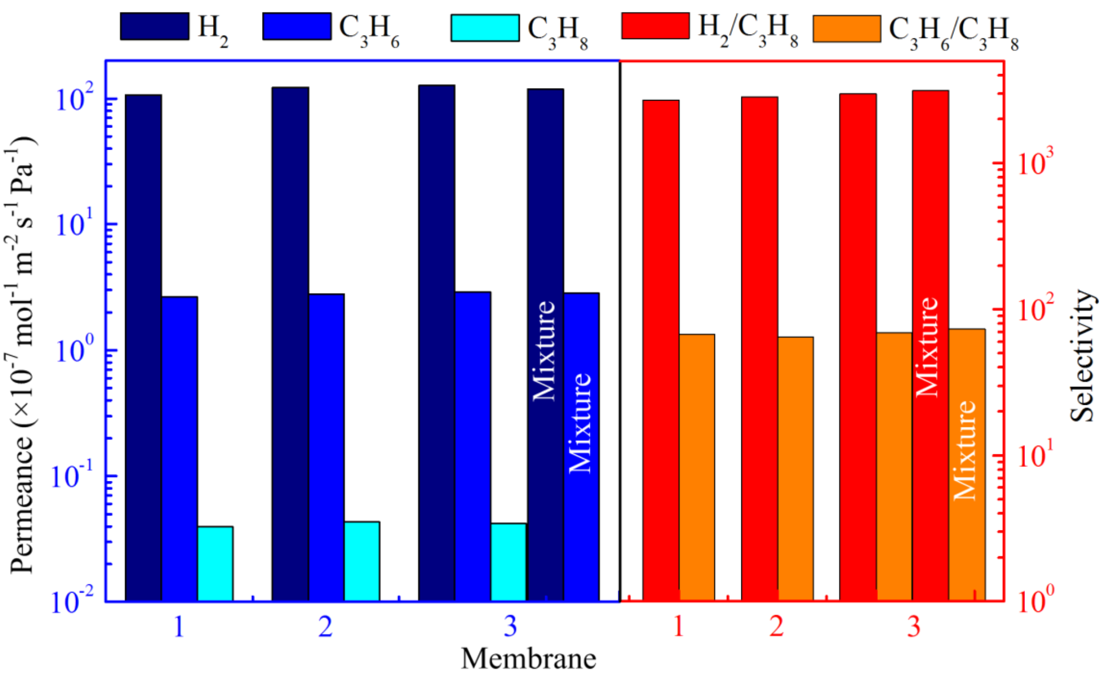

Supplementary Figure 11 | Gas permeation behaviours of additional three ZIF-8 membranes. These ZIF-8 membranes were prepared with sol concentration of 1 U, coating time of 2 s and deposition time of 2 h. The H_2 permeances of these three membranes were 106.5, 122.9 and 126.7 $\times10^{-7}$ mol m^{-2} s^{-1} Pa^{-1}, and the corresponding selectivities of H_2/C_3H_8-C_3H_6/C_3H_8 were 2701-67.2, 2845-64.4 and 3012-68.9, respectively. The results demonstrate the good reproducibility of the membranes. In binary mixture separation of additional ZIF-8 membrane 3, the similar permeances (117.8 $\times10^{-7}$ mol m^{-2} s^{-1} Pa^{-1} for H_2 in H_2/C_3H_8 separation and 2.8 $\times10^{-7}$ mol m^{-2} s^{-1} Pa^{-1} for C_3H_6 in C_3H_6/C_3H_8 separation) and selectivities (3126 for H_2/C_3H_8 and 73.4 for C_3H_6/C_3H_8) were achieved.

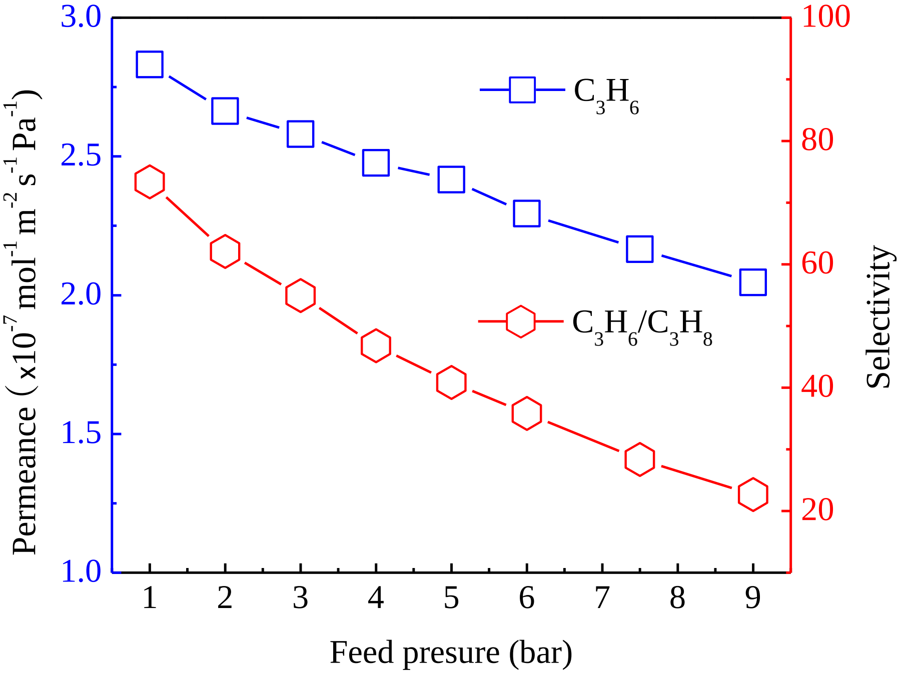

Supplementary Figure 12 | Effect of feed pressure on separation performance of C_3H_6/C_3H_8 mixture. The membrane was the additional ZIF-8 membrane 3, as shown in Supplementary Figure 11. The selectivity and the C_3H_6 permeance both decreased with the increase of feed pressure due to the gate opening of window of ZIF-8 structure and the competitively diffusion of the C_3H_6 and C_3H_8 through the membrane. However, membrane still showed good C_3H_6/C_3H_8 selectivity of 22.7 and C_3H_6 permeance of 2.0 $\times 10^{-7}$ mol m^{-2} s^{-1} Pa^{-1} at 9 bar.

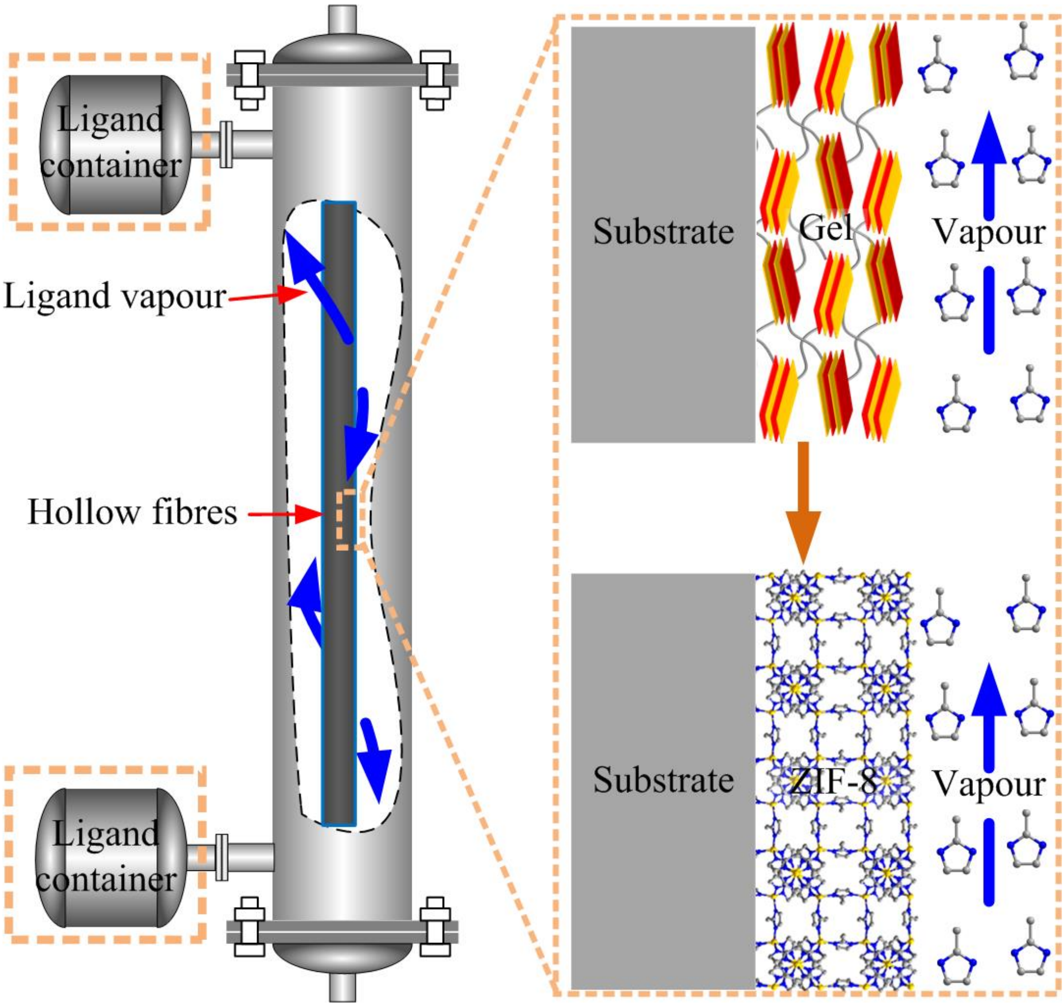

Supplementary Figure 13 | Schematic diagram of in situ production of MOF membrane module (left) and crystallization process (right). Blue arrows present the diffusion of ligand vapour.

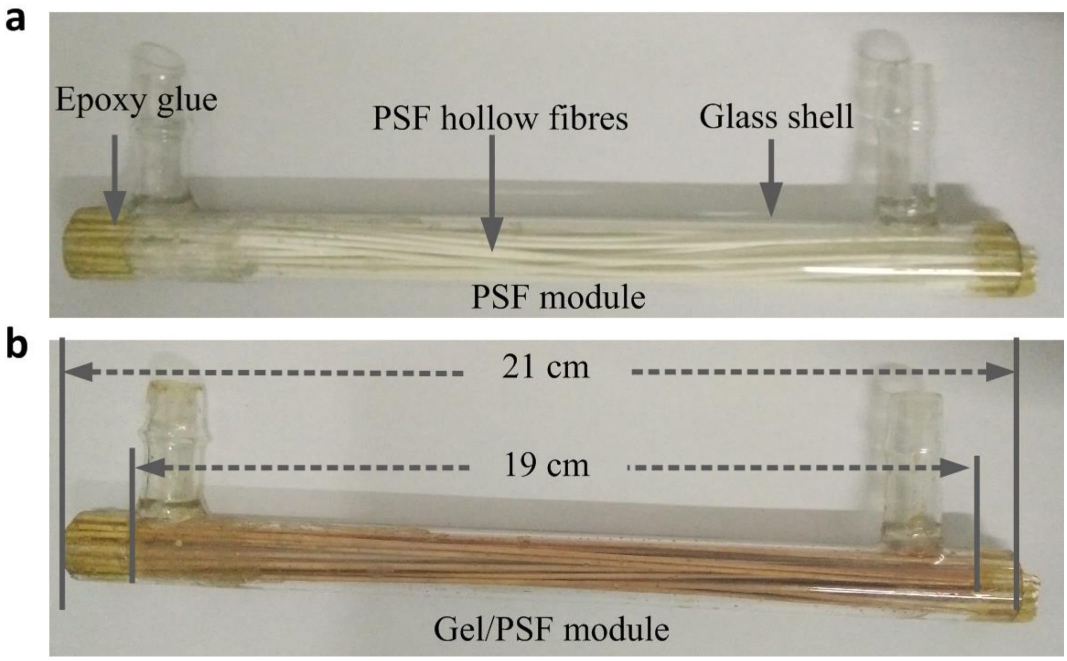

Supplementary Figure 14 | Optical photographs of membrane module and coated membrane module. **a**, membrane module and **b**, coated membrane module. The change of colour should be explained by the reaction between polymer and ethanolamine.

Supplementary Table 1 | Comparison of ZIF-8 membranes here with polymeric, zeolite and other MOF membranes reported in previous studies for H_2/N_2, H_2/CH_4 and H_2/C_3H_8 systems.

Membrane	Substrate	Thickness (100 nm)	Permeance $\times10^{-8}$ mol m^{-2} s^{-1} Pa^{-1}	Selectivity			Ref
				H_2/N_2	H_2/CH_4	H_2/C_3H_8	
ZIF-8	Al_2O_3	80	20.8	10.3	10.4	149.6	1
ZIF-8	Al_2O_3	200	18.0	16.2	31.5	712.6	2
ZIF-8	Al_2O_3	20	43.2	11.1	12.1	-	3
ZIF-8/LDH	Al_2O_3	200	14	10	12.5	-	4
ZIF-8/LDH	Al_2O_3	11	4.1	16.8	54.1	-	5
ZIF-8	PES	72	40.0	9.2	8.7	-	6
ZIF-8	PSF	200	11.1	22.7	-	-	7
ZIF-8-annealing	P84	13	3.5	-	103	-	8
ZIF-93-annealing	P84	26	1.10	-	97.2	-	9
ZIF-8/GO	AAO	1	5.46	11.1	11.2	405.0	10
ZIF-90-modification	Al_2O_3	200	28.5	-	70.5	458.0	11
ZIF-95	Al_2O_3	300	193.0	10.1	11.0	59.7	12
NH_2-MIL-53	Glass	150	151.7	23.9	20.7	-	13
CuBTC	Al_2O_3	130	3.2	8.7	6.2	-	14
CuBTC	PVDF	30	201.0	6.5	5.4	-	15
CuBTC/MIL-100	PVDF	200	8.8	217	336	-	16
PIM	-	1810	1.4	14.8	11.1	-	17
PIM	-	1570	0.5	9.4	4.9	-	17
Zeolite	Al_2O_3	75	16	8.6	6.5	19.3	18
ZIF-8	PVDF	0.17	2154	15.1	17.9	1145	This work
ZIF-8	PVDF	0.87	1190	22.4	27.3	2894	
ZIF-8	PVDF	1.14	949	23.1	28.7	3088	
ZIF-8	PSF	2.80	354	24.1	30.7	3212	
ZIF-8	PVDF	7.57	273	24.8	31.2	3401	

Supplementary Table 2 | Comparison of ZIF-8 membranes here with carbon and other MOF membranes reported in previous studies for C_3H_6/C_3H_8 system.

Membrane	Substrate	Thickness (100 nm)	Permeance $\times10^{-8}$ mol m^{-2} s^{-1} Pa^{-1}	Selectivity C_3H_6/C_3H_8	Ref
ZIF-8	Al_2O_3	22	2.0-3.7	28.0-45.0	19
ZIF-8	Al_2O_3	15	2.0	55.0	20
ZIF-8	Al_2O_3	50-200	0.7	30.0	21
ZIF-8	Al_2O_3	800	0.3	59.0	22
ZIF-8	Al_2O_3	15	2.0	40.0	23
ZIF-8	Al_2O_3	25	1.1	30.1	24
ZIF-8	Al_2O_3	200	0.35	14.6	3
ZIF-8	Al_2O_3	16	0.06	3.5	25
ZIF-8/GO	AAO	1	0.16	12	10
ZIF-8	BPPO	20	1.5	27.8	26
ZIF-8	Torlon	90	1.2	12.0	27
ZIF-8	Torlon	50	2.2	65	28
ZIF-8	Torlon	81	1.5	180	29
Cabron	Al_2O_3	3	0.75	16	30
Cabron	Al_2O_3	3	0.87	17	30
Cabron	Al_2O_3	3	1.4	23	30
ZIF-8	PVDF	0.17	83.7	44.5	This work
ZIF-8	PVDF	0.87	27.6	67.2	
ZIF-8	PVDF	1.14	20.8	67.8	
ZIF-8	PSF	2.80	7.6	69.5	
ZIF-8	PVDF	7.57	5.7	70.8	

Supplementary References

1. Zhang, X. F. *et al*. A simple and scalable method for preparing low-defect ZIF-8 tubular membranes. *J. Mater. Chem. A* **1**, 10635-10638 (2013).
2. Liu, Q., Wang, N., Caro, J. & Huang, A. Bio-inspired polydopamine: A versatile and powerful platform for covalent synthesis of molecular sieve membranes. *J. Am. Chem. Soc.* **135**, 17679-17682 (2013).
3. Huang, K., Dong, Z. Y., Li, Q. Q. & Jin, W. Q. Growth of a ZIF-8 membrane on the inner-surface of a ceramic hollow fiber via cycling precursors. *Chem. Commun.* **49**, 10326-10328 (2013).
4. Liu, Y., Wang, N. Y., Pan, J. H., Steinbach, F. & Caro, J. In-situ synthesis of MOF membranes on ZnAl-CO_3 LDH buffer layer-modified substrates. *J. Am. Chem. Soc.* **136**, 14353-14356 (2014).
5. Liu, Y. *et al*. Remarkably enhanced gas separation by partial self-conversion of a laminated membrane to metal-organic frameworks. *Angew. Chem. Int. Ed.* **54**, 3028-3032 (2015).
6. Ge, L., Zhou, W., Du, A. J. & Zhu, Z. H. Porous polyethersulfone-supported zeolitic imidazolate framework membranes for hydrogen separation. *J. Phys. Chem. C* **116**, 13264-13270 (2012).
7. Su, P. *et al*. Metal based gels as versatile precursors to synthesize stiff and integrated MOF/polymer composite membranes. *J. Mater. Chem. A* **3**, 20345-20351 (2015).
8. Cacho-Bailo, F. *et al*. MOF-polymer enhanced compatibility: post-annealed zeolite imidazolate framework membranes inside polyimide hollow fibers. *RSC Adv.* **6**, 5881-5889 (2016).

9. Cacho-Bailo, F. *et al*. High selectivity ZIF-93 hollow fiber membranes for gas separation. *Chem. Commun.* **51**, 11283-11285 (2015).

10. Hu, Y. X. *et al*. Zeolitic imidazolate framework/graphene oxide hybrid nanosheets as seeds for the growth of ultrathin molecular sieving membranes. *Angew. Chem. Int. Ed.* **55**, 204-2052 (2016).

11. Huang, A., Wang, N., Kong, C. & Caro, J. Organosilica-functionalized zeolitic imidazolate framework ZIF-90 membrane with high gas-separation performance. *Angew. Chem. Int. Ed.* **51**, 10551-10555 (2012).

12. Huang, A. *et al*. A highly permeable and selective zeolitic imidazolate framework ZIF-95 membrane for H_2/CO_2 separation. *Chem. Commun.* **48**, 10981-10983 (2012).

13. Zhang, F. *et al*. Hydrogen selective NH_2-MIL-53(Al) MOF membranes with high permeability. *Adv. Funct. Mater.* **22**, 3583-3590 (2012).

14. Zhou, S. Y. *et al*. Challenging fabrication of hollow ceramic fiber supported $Cu_3(BTC)_2$ membrane for hydrogen separation. *J. Mater. Chem.* **22**, 10322-10328 (2012).

15. Mao, Y. Y. *et al*. Pressure-assisted synthesis of HKUST-1 thin film on polymer hollow fiber at room temperature toward gas separation. *ACS Appl. Mater. Interfaces* **6**, 4473-4479 (2014).

16. Li, W. *et al*. Transformation of metal-organic frameworks for molecular sieving membranes. *Nature Commun.* **7**, 11315 (2016).

17. Carta, M. *et al*. An efficient polymer molecular sieve for membrane gas separations. *Science* **339**, 303-307 (2013).

18. Huang, A., Wang, N. & Caro, J. Synthesis of multi-layer zeolite LTA membranes with enhanced gas separation performance by using 3-aminopropyltriethoxysilane as interlayer.

Microporous Mesoporous Mater. **164**, 294-301 (2012).

19. Pan, Y., Li, T., Lestari, G. & Lai, Z. Effective separation of propylene/propane binary mixtures by ZIF-8 membranes. *J. Membr. Sci.* **390**, 93-98 (2012).

20. Kwon, H. T. & Jeong, H. K. In situ synthesis of thin zeolitic-imidazolate framework ZIF- 8 membranes exhibiting exceptionally high propylene/propane separation. *J. Am. Chem. Soc.* **135**, 10763-10768 (2013).

21. Shah, M. N., Gonzalez, M. A., McCarthy, M. C. & Jeong, H. K. An unconventional rapid synthesis of high performance metal-organic framework membranes. *Langmuir* **29**, 7896-7902 (2013).

22. Hara, N. *et al*. Diffusive separation of propylene/propane with ZIF-8 membranes. *J. Membr. Sci.* **450**, 215-223 (2014).

23. Kwon, H. T. & Jeong, H. K. Highly propylene-selective supported zeolite-imidazolate framework (ZIF-8) membranes synthesized by rapid microwave-assisted seeding and secondary growth. *Chem. Commun.* **49**, 3854-3856 (2013).

24. Liu, D., Ma, X., Xi, H. & Lin, Y. S. Gas transport properties and propylene/propane separation characteristics of ZIF-8 membranes. *J. Membr. Sci.* **451**, 85-93 (2014).

25. Shekhah, O. *et al*. The liquid phase epitaxy approach for the successful construction of ultra-thin and defect-free ZIF-8 membranes: pure and mixed gas transport study. *Chem. Commun.* **50**, 2089-2092 (2014).

26. Shamsaei, E. *et al*. Aqueous phase synthesis of ZIF- 8 membrane with controllable location on an asymmetrically porous polymer substrate. *ACS Appl. Mater. Interfaces* **8**, 6236-6244 (2016).

27. Brown, A. J. *et al*. Interfacial microfluidic processing of metal-organic framework hollow fiber membranes. *Science* **345**, 72-75 (2014).

28. Eum, K. *et al*. Fluidic processing of high-performance ZIF-8 membranes on polymeric hollow fibers: Mechanistic insights and microstructure control. *Adv. Funct. Mater.* **26**, 5011-5018 (2016).

29. Eum, K., Ma, C., Rownaghi, A., Jones, C. W. & Nair, S. ZIF- 8 membranes via interfacial microfluidic processing in polymeric hollow fibers: efficient propylene separation at elevated pressures. *ACS Appl. Mater. Interfaces* **8**, 25337-25342 (2016).

30. Ma, X., Lin, Y. S., Wei, X. & Kniep, J. Ultrathin carbon molecular sieve membrane for propylene/propane separation. *AIChE J*. **62**, 491-499 (2016).
